# Heparan sulfate is necessary for the early formation of nascent fibronectin and collagen I fibrils at matrix assembly sites

**DOI:** 10.1016/j.jbc.2021.101479

**Published:** 2021-12-07

**Authors:** Katherine E. Hill, Benjamin M. Lovett, Jean E. Schwarzbauer

**Affiliations:** Department of Molecular Biology, Princeton University, Princeton, New Jersey, USA

**Keywords:** fibronectin, heparan sulfate, heparin, matrix assembly, glycosyltransferase, BCS, bovine calf serum, CHO, Chinese hamster ovary, DMEM, Dulbecco’s Modified Eagle’s Medium, DOC, deoxycholate detergent, DTDST, diastrophic dysplasia sulfate transporter, ECM, extracellular matrix, EXT1, exostosin-1, EXT2, exostosin-2, FN, fibronectin, GAG, glycosaminoglycans, GAPDH, glyceraldehyde 3-phosphate dehydrogenase, HME, hereditary multiple exostoses, HS, heparan sulfate, HSPG, heparan sulfate-modified proteoglycan

## Abstract

Fibronectin (FN), an essential component of the extracellular matrix (ECM), is assembled *via* a cell-mediated process in which integrin receptors bind secreted FN and mediate its polymerization into fibrils that extend between cells, ultimately forming an insoluble matrix. Our previous work using mutant Chinese hamster ovary (CHO) cells identified the glycosaminoglycan heparan sulfate (HS) and its binding to FN as essential for the formation of insoluble FN fibrils. In this study, we investigated the contributions of HS at an early stage of the assembly process using knockdown of exostosin-1 (EXT1), one of the glycosyltransferases required for HS chain synthesis. NIH 3T3 fibroblasts with decreased EXT1 expression exhibited a significant reduction in both FN and type I collagen in the insoluble matrix. We show that FN fibril formation is initiated at matrix assembly sites, and while these sites were formed by cells with EXT1 knockdown, their growth was stunted compared with wild-type cells. The most severe defect observed was in the polymerization of nascent FN fibrils, which was reduced 2.5-fold upon EXT1 knockdown. This defect was rescued by the addition of exogenous soluble heparin chains long enough to simultaneously bind multiple FN molecules. The activity of soluble heparin in this process indicates that nascent fibril formation depends on HS more so than on the protein component of a specific HS proteoglycan. Together, our results suggest that heparin or HS is necessary for concentrating and localizing FN molecules at sites of early fibril assembly.

Assembly of a fibronectin (FN) extracellular matrix (ECM) is essential for embryogenesis and the development, repair, regeneration, and homeostasis of all tissues. Disrupted or disordered fibronectin assembly occurs in many diseases and is key to the progression of fibrosis and scarring. FN binding to integrin receptors, especially α5β1 integrin, is required for assembly (1, 2). Fibril formation also depends on FN self-association, mediated by its N-terminal assembly domain (3). FN has many other binding partners, including the glycosaminoglycans (GAGs) heparan sulfate and heparin that interact with its main heparin-binding domain (HepII) (4). A dose-dependent loss of FN matrix was detected when GAG chain addition to proteins was blocked with xylosides (5) further implicating GAGs in matrix assembly.

Heparan sulfate (HS)-modified proteoglycans (HSPGs) are found within the ECM and at the cell surface. HS chains are elongated onto core proteins in the Golgi apparatus by a complex of glycosyltransferases, exostosin-1 (EXT1) and exostosin-2 (EXT2) (6, 7, 8, 9, 10). Screening of a mutagen-treated Chinese hamster ovary (CHO) cell library identified CHO-677 cells, which show significantly reduced EXT1 mRNA levels and GlcA/GlcNAc transferase activities compared with wild-type CHO cells. The mutation eliminates HS synthesis (11) and causes a severe deficiency in assembly of FN (12, 13) presumably due to decreased EXT1 expression although the specific mutation has not been determined. Mutations in EXT1 have been linked to skeletal abnormalities and osteochondromas (14). Conditional knockout of EXT1 in limb bud mesenchyme causes defects in growth and differentiation of cartilage condensations, including an abnormal perichondrium with dispersed FN matrix (15). It seems clear that HS plays a role in FN matrix assembly and organization both *in vivo* and in cell culture.

Our previous work using CHO cells identified a role for HS in conversion of FN fibrils into a stabilized, detergent-insoluble form (13). Since insolubility is the final step in FN assembly, this finding shows that HS functions late in assembly. We also observed higher levels of detergent-soluble FN in lysates from CHO cells treated with heparin. This observation suggests that HS may have another role in assembly, in an earlier step. To determine how HS promotes FN fibrillogenesis in cells that normally produce and assemble FN, we performed siRNA-mediated knockdown of EXT1 in NIH 3T3 fibroblasts and investigated the effects of loss of HS on the progression of FN matrix assembly. We analyzed and quantified matrix assembly sites (the initial sites of FN fibril formation), nascent FN fibrils, and stable FN matrix in mock treated *versus* EXT1 knockdown fibroblasts. Here we show that decreasing EXT1 mRNA expression in fibroblasts reduces HS production causing defects in the initial stages of FN fibrillogenesis. Furthermore, the role of HS/heparin is chain length dependent. We propose that in addition to its role in forming insoluble FN fibrils, HS binding to FN is also involved in early steps of assembly where it promotes FN-FN interactions by concentrating FN near sites of matrix assembly initiation.

## Results

### Reduced HS with EXT1 knockdown in NIH 3T3 cells

**Figure 1:**
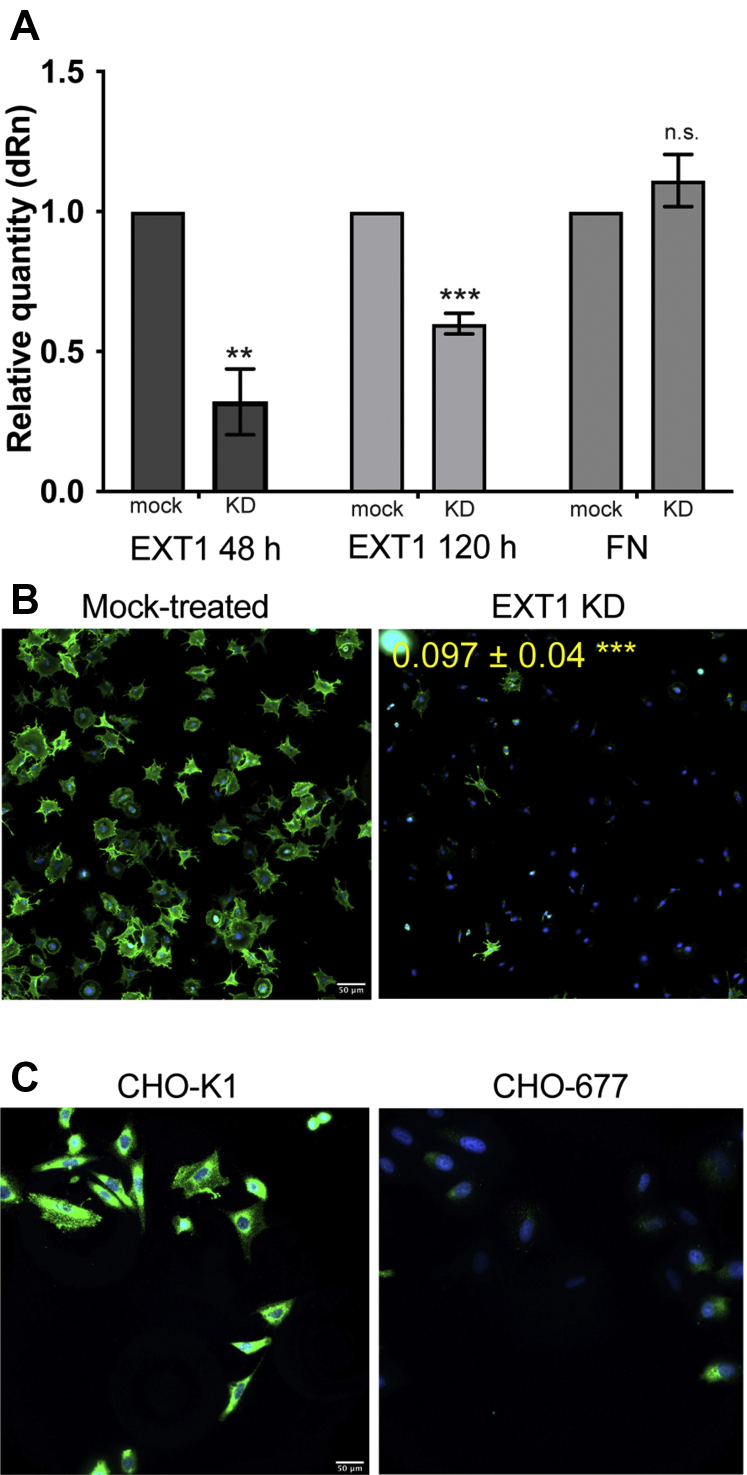
**Reduced HS with EXT1 knockdown in fibroblasts.***A*, RNA was isolated from EXT1 siRNA-treated (KD) and mock-transfected NIH 3T3 fibroblasts at 48 and 120 h after transfection. Expression of EXT-1 and FN (at 48 h) was assessed by qPCR. Each sample was normalized to GAPDH, and fold-change was calculated relative to mock-treated cells. Error bars represent SEM. ∗∗*p* < 0.01, ∗∗∗*p* < 0.001, n.s. = not significant (three independent experiments). *B*, mock-treated and EXT-1 knockdown cells were grown on FN-coated coverslips for 4 h and then fixed and stained with anti-HS antibody (*green*) and DAPI (*blue*). Scale = 50 μm. Representative images are shown for each condition. ∗∗∗*p* < 0.001. *C*, wild-type Chinese hamster ovary cells (CHO-K1) and HS-deficient CHO-677 mutant cells grown on FN-coated coverslips for 4 h were fixed and stained with anti-HS antibody (*green*) and DAPI (*blue*). Scale = 50 μm. Representative images are shown for each cell type.

To establish the role of HS in assembly of an FN matrix, we used NIH 3T3 fibroblasts, which synthesize and secrete high levels of FN and assemble it into a dense fibrillar matrix. NIH 3T3 fibroblasts were treated with an siRNA SMARTpool targeting the EXT1 transcript. Analysis of RNA harvested 48 h and 120 h posttransfection shows that, compared with mock-transfected cells, EXT1 mRNA was significantly decreased with siRNA knockdown by approximately 60% at 48 h and 40% at 120 h as determined by qPCR (Fig. 1*A*). Since EXT1 mRNA levels appear to begin recovering at 120 h, all experiments were conducted within 4 days of siRNA treatment. FN transcript levels were measured in parallel and did not show a significant change (Fig. 1*A*). In addition, there was no significant difference in α5 integrin levels between mock and EXT1 knockdown cells (data not shown). Therefore, any difference in FN matrix assembly is not due to loss of FN or its primary receptor.

EXT1 is required for synthesis of HS so we evaluated HS levels by immunofluorescence staining. HS fluorescence was significantly reduced, ∼tenfold, for cell surface HS detected with an anti-HS monoclonal antibody (10E4) in EXT1 knockdown cells compared with mock-treated cells (Fig. 1*B*). A similar difference in anti-HS staining was observed between wild-type CHO cells and HS-deficient CHO-677 cells (Fig. 1*C*). These results confirm that siRNA knockdown of EXT1 significantly reduced HS expression.

### EXT1 knockdown cells assemble less FN matrix

**Figure 2:**
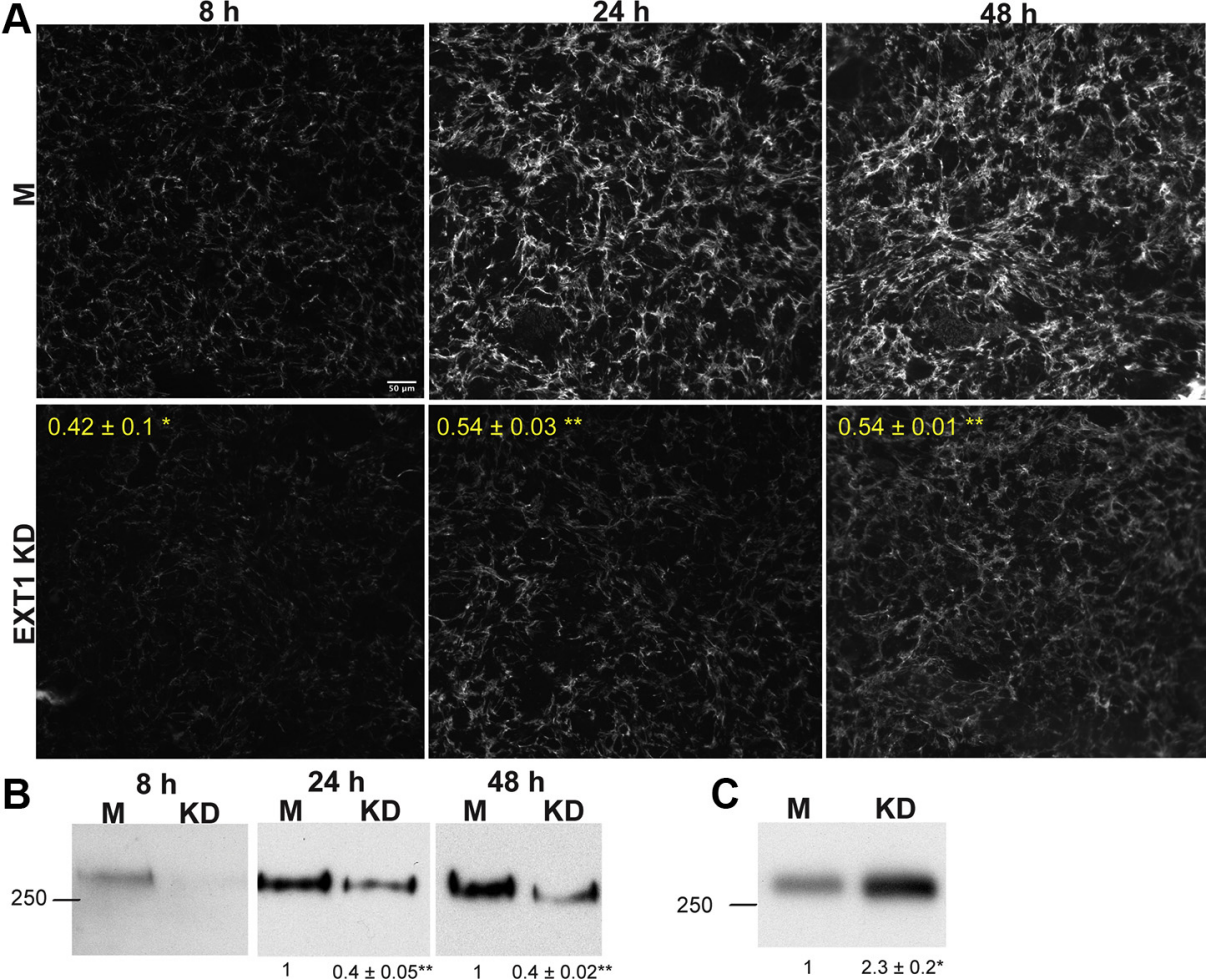
**EXT1 knockdown decreases FN matrix.***A*, confluent EXT1 knockdown (KD) and mock (M) NIH 3T3 fibroblasts were fixed and immunostained for FN at the indicated times. Mean fluorescence intensities from three independent experiments were calculated, and average fold differences were determined between KD and M samples, shown in *yellow*. Scale = 50 μm. *B*, DOC-insoluble matrix fractions were isolated from DOC cell lysates at the indicated times. Samples were separated on a 6% polyacrylamide–SDS gel, transferred, and immunoblotted with an anti-FN antiserum. Fold-differences for KD compared with M were calculated from band intensities, normalized to GAPDH levels in the DOC-soluble fraction (Fig. S1), and are the average of three independent experiments. Position of 250 kD molecular mass marker is shown (In order to see the FN band in 8 h M lane, blot was exposed three times longer than the 24 h and 48 h samples). *C*, conditioned media were collected after culturing KD and M fibroblasts for 96 h in FN-depleted serum. Samples were immunoblotted with anti-FN antiserum, bands were quantified, and fold differences were calculated as above (*A*–*C*, ∗*p* < 0.05, ∗∗*p* < 0.01).

To test if decreased EXT1 and HS lead to a decrease in FN matrix assembly, matrix levels were assessed by immunofluorescence at 8 h, 24 h, and 48 h postseeding. Mock-treated cells assembled more FN matrix than EXT1 knockdown cells at all time points. Mean fluorescence intensities of FN matrix were significantly lower in EXT1 knockdown cells in comparison to the mock-transfected cells (Fig. 2*A*).

Nascent FN fibrils are soluble in deoxycholate (DOC) detergent but are ultimately converted to a DOC-insoluble form, which serves as a foundational matrix for deposition of other ECM proteins (1). Analysis of DOC-insoluble matrix provides a quantitative measure of matrix assembly. Immunoblots of DOC-insoluble FN showed dramatically higher FN levels in mock-treated compared with EXT1 knockdown cells at 8, 24, and 48 h (Fig. 2*B*). Quantification of DOC-insoluble FN normalized to GAPDH (Fig. S1) shows that FN levels in EXT1 knockdown cells never reach the level detected in mock cells even at the latest time point indicating that decreased matrix is not due to a simple difference in the rate of FN assembly. We conclude that HS facilitates assembly of stable matrix and, in its absence, assembly is defective. In support of this conclusion, we found ∼2.3-fold higher FN in the conditioned medium of EXT1 knockdown cells compared with mock-transfected cells suggesting that, while similar levels of FN are produced (as shown by qPCR in Fig. 1*A*), less FN is assembled in the absence of EXT1.

**Figure 3:**
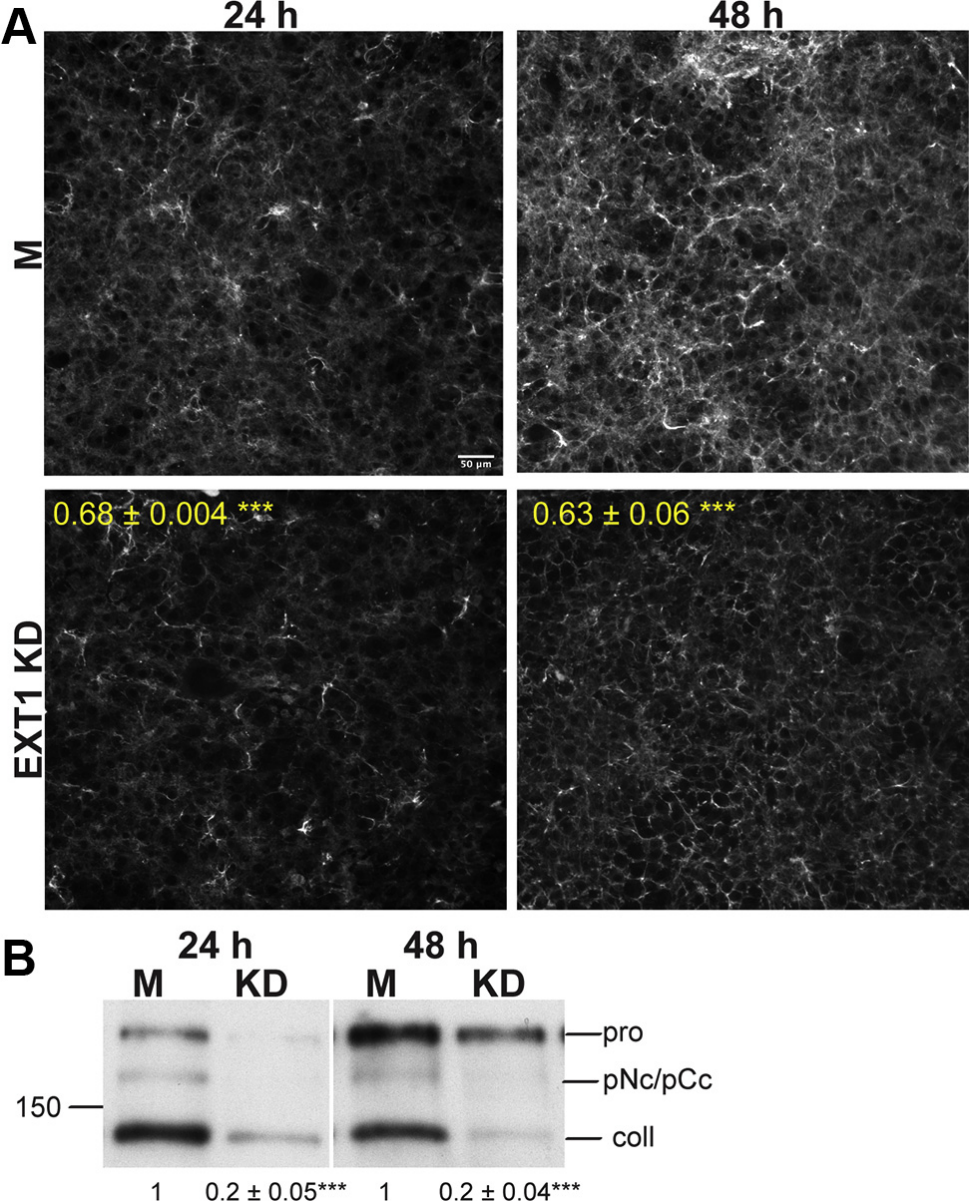
**EXT1 knockdown decreases type I collagen matrix.***A*, EXT1 knockdown (KD) and mock (M) cells were fixed and immunostained for type I collagen at the indicated times. Mean fluorescence was quantified as in Figure 2*A*. Scale bar = 50 μm. *B*, DOC-insoluble matrix fractions were separated on a 5% polyacrylamide–SDS gel, transferred, and immunoblotted with anti-collagen I antibody. Fold-differences between M and KD fully processed collagen bands were calculated as in Figure 2*B*. Locations of procollagen, pN- or pC-collagen, and fully processed collagen I bands are indicated (*right*). Position of 150 kD molecular mass marker is on the *left* (*A* and *B*, ∗∗∗*p* < 0.001; three independent experiments).

The genetic bone disorder, hereditary multiple exostoses (HME), is caused by mutations in EXT1 and is characterized by multiple benign bony outgrowths (16). Type I collagen, the major protein component of bones, is critical for proper bone development and structure (17). Since an FN matrix is necessary for collagen I deposition (18, 19, 20, 21), we examined if type I collagen levels were decreased in EXT1 knockdown fibroblasts *via* immunofluorescence. We saw a significant decrease of ∼30 to 40% in type I collagen fibrils at 24 h and 48 h (Fig. 3*A*). Procollagen is secreted into the extracellular space where its N- and C-propeptides are cleaved by proteinases to promote collagen fibrillogenesis (22). Analysis of DOC-insoluble matrix showed three bands—fully processed collagen being the most predominant band at ∼140 kD, and two bands at ∼200 kD and ∼170 kD representing procollagen and partially processed pNcollagen or pCcollagen, respectively (Fig. 3*B*) (21). All three proteins were reduced in the EXT1 knockdown cells, but fully processed collagen showed the most drastic decrease, with an approximately 80% reduction at both time points. These findings show that EXT1 and HS are also required for formation of insoluble collagen fibrils.

Matrix assembly could be reduced if cell adhesion was affected by EXT1 knockdown. Comparison of mock-treated and knockdown cells showed no significant difference in attachment across a range of FN coating concentrations (Fig. S2*A*). We also assessed cell spreading after 2 h on a FN substrate and found that mock-treated and knockdown cells had similar average cell areas (Fig. S2*B*). Thus the reduction in FN matrix with EXT1 knockdown is not due to defects in adhesion and spreading.

### HS promotes matrix assembly site formation

**Figure 4:**
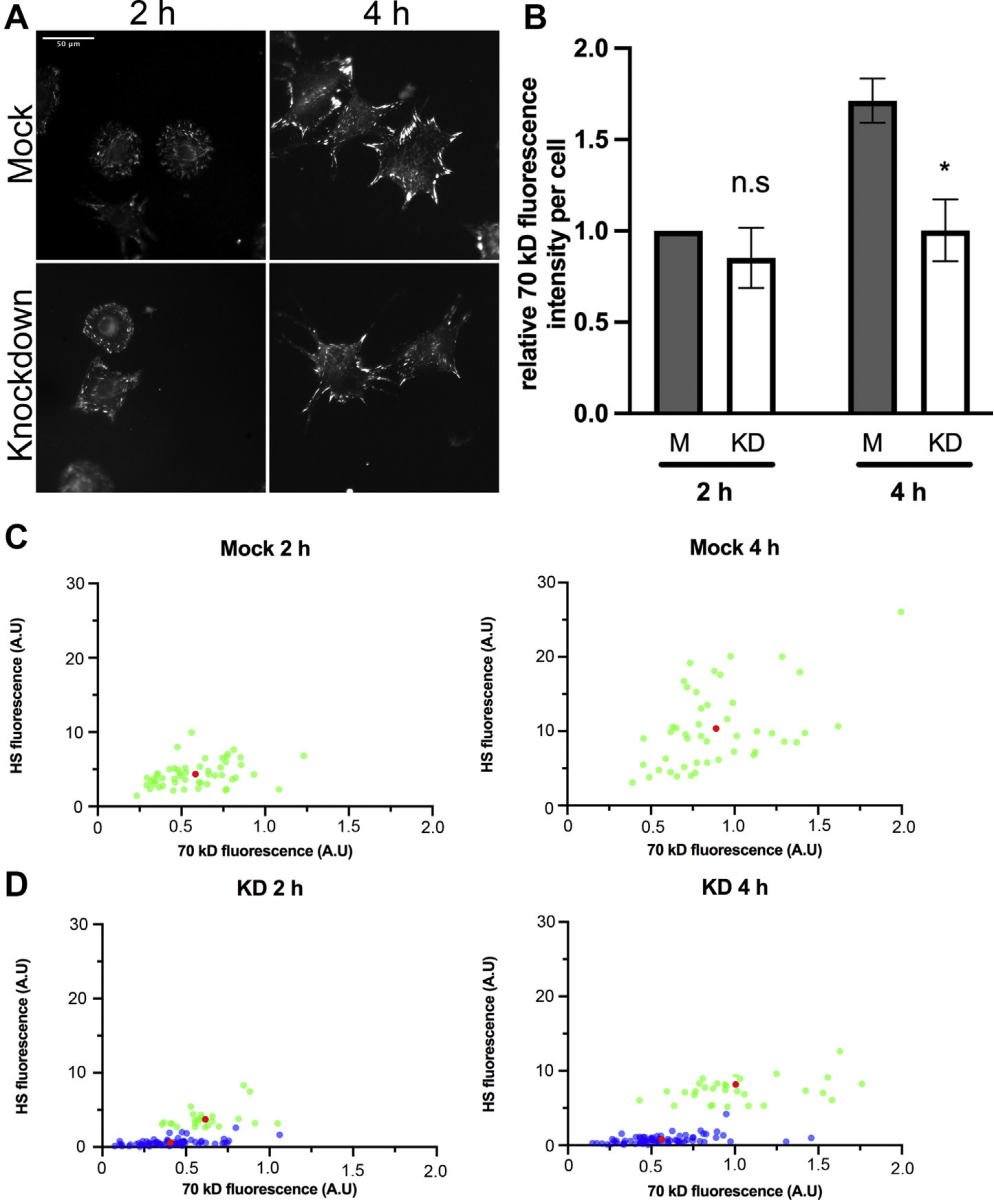
**Loss of HS changes matrix assembly site formation.** Mock and EXT1 knockdown cells were grown on FN-coated coverslips for 2 or 4 h. Matrix assembly sites were detected with biotinylated 70 kD and fluorescent-streptavidin. *A*, representative images of matrix assembly sites are shown. Scale = 50 μm. *B*, mean fluorescence intensities per cell were quantified for three independent experiments and normalized to 2 h mock cells. Significance tests were performed between M and KD at both time points. ∗*p* < 0.05, n.s. = not significant. Mock (*C*) and KD (*D*) cells were costained for matrix assembly sites and HS at 2 and 4 h. Each point represents the fluorescence intensities for a single cell in arbitrary units (a.u.). Graphs shown are representative of the fluorescence intensities of three separate experiments. A *red dot* indicates the mean of the *green* or *blue dot* population. *D*, *green dots* represent high-HS staining cells with a HS fluorescence within one SD of the mock-treated mean HS fluorescence. *Blue dots* represent low-HS staining cells with HS fluorescence greater than one standard deviation below the mock-treated mean HS fluorescence.

The effect of EXT1 knockdown on fibril density and insolubility resembles our results with CHO-677 mutant cells (13) and further demonstrates that HS is necessary for formation of irreversible, insoluble FN matrix. It remains possible that HS also acts earlier in the assembly process so we examined whether HS has a role in formation of matrix assembly sites, the initial sites of FN fibril nucleation. Matrix assembly sites form through FN conformational changes induced when integrins pull on surface-adsorbed FN exposing binding sites for the N-terminal assembly domain of FN (23). Biotin-tagged 70 kD N-terminal fragment of FN, which contains the assembly domain, and fluorescently-tagged streptavidin were used to visualize matrix assembly sites by fluorescence microscopy. 70 kD fluorescence intensity of individual cells was quantified, and the average fluorescence intensity per cell for each population was used as a measure of matrix assembly site growth. At 2 h, both mock-treated and knockdown cells had very small sites of 70 kD localization (Fig. 4*A*) with no significant difference in average intensities (Fig. 4*B*). However, by 4 h, 70 kD binding to sites on mock-treated cells was significantly higher than binding to knockdown cells by ∼1.7-fold (Fig. 4, *A* and *B*). This finding shows that matrix assembly sites grow more slowly in the absence of EXT1 and HS.

A small portion of cells (∼5%) within the EXT1 knockdown population displayed dramatically higher levels of HS staining than the rest of the population. We examined whether this higher level of HS staining correlated with higher 70 kD labeling of matrix assembly sites. HS fluorescence of individual cells from the mock and knockdown populations was measured and plotted *versus* 70 kD fluorescence of matrix assembly sites (Fig. 4, *C* and *D*). Between 2 and 4 h, the average HS fluorescence per mock-treated cell approximately doubled (Table S1) and the 70 kD labeling increased by ∼1.6-fold (Fig. 4*C* and Table S2). In contrast, the knockdown cells could be divided into two populations using the means and standard deviations of the HS fluorescence signals in the mock populations. Those knockdown cells with HS fluorescence within one standard deviation of the mock cell mean fluorescence intensity at the same time point were designated as having “high-HS” (green dots, Fig. 4*D*). Remaining knockdown cells where the HS fluorescence was greater than one standard deviation below the mock cell mean fluorescence intensity were designated as “low-HS” (blue dots, Fig. 4*D*). The HS fluorescence intensity, averaged over four separate experiments, differed by almost tenfold between these two populations (Fig. 4*D* and Table S1). The average 70 kD fluorescence intensity of high-HS knockdown cells closely resembled that of mock-treated cells at both 2 and 4 h time points (compare green populations in panels Fig. 4, *C* and *D*). However, the low-HS cells had significantly lower 70kD fluorescence intensity than the high-HS cells in the same population (∼1.9-fold at both 2 h and 4 h, Table S2). The difference in fluorescence intensity between the high-HS and low-HS populations did not change from 2 to 4 h, showing that the growth of matrix assembly sites in the low-HS group is not catching up with the high-HS group over time. Therefore, the loss of HS is causing a severe deficiency in matrix assembly site formation rather than simply delaying their growth. These results strengthen our conclusion that matrix assembly site development depends on HS.

To explore if there is a general recruitment of FN to the cell surface independent of matrix assembly, FN binding to mock-treated and EXT1 knockdown fibroblasts was measured using cells attached to tissue culture plastic, a substrate that does not promote matrix assembly site formation, and using cells in suspension. No significant difference in FN binding was detected in either assay (data not shown). We conclude that cell surface localization of FN by HS depends on the establishment of matrix assembly sites.

### HS/heparin promotes nascent FN fibril formation

**Figure 5:**
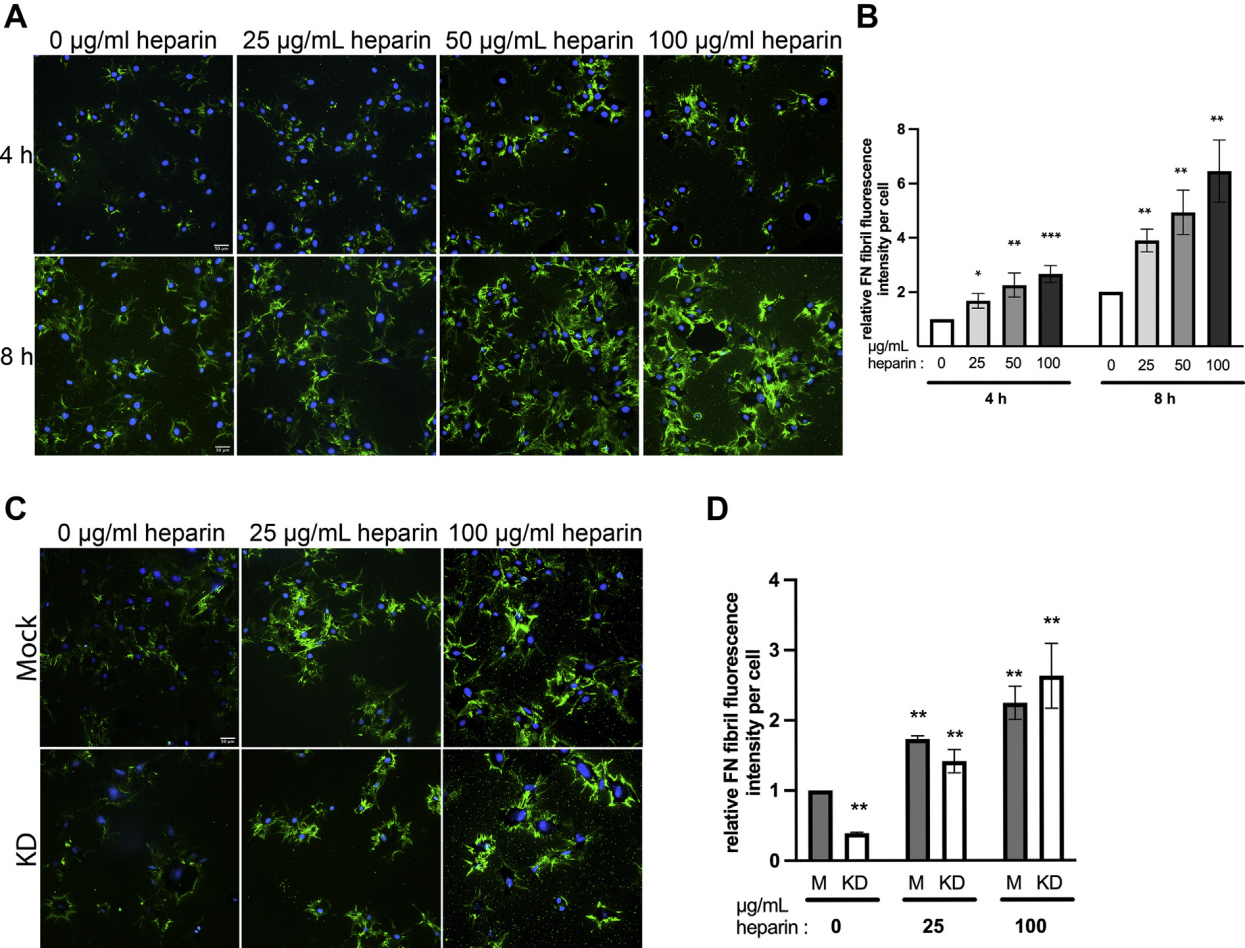
**Exogenous heparin increases nascent FN fibril assembly.** NIH 3T3 (*A*) or mock and KD (*C*) cells were grown on rat FN-coated coverslips in medium containing 25 μg/ml human FN and increasing concentrations of heparin. Cells were fixed and stained with anti-human FN-specific antibody hFN7.1 (*green*) and DAPI (*blue*). Scale = 50 μm. Representative images are shown for each condition. FN fibril fluorescence intensity per cell was quantified at 4 and 8 h and expressed relative to 4 h–0 heparin treatment set to 1 (*B*). Mock and KD cells at 8 h were normalized to M cells–0 heparin treatment set to 1 (*D*). Significance was determined for each treatment compared with 0 heparin in the same group. ∗*p* < 0.05, ∗∗*p* < 0.01, ∗∗∗*p* < 0.001, three independent experiments. *D*, the difference between M and KD pairs at either 25 or 100 μg/ml heparin treatment is not significant (*p* > 0.05).

With time, matrix assembly sites develop into fibrillar adhesions and nascent fibrils that are ultimately elaborated into an insoluble fibrillar matrix (24). We monitored steps in fibrillogenesis that follow matrix assembly site formation by providing subconfluent NIH 3T3 cells with exogenous FN and increasing concentrations of heparin. FN fibrils were evaluated by FN immunostaining and quantification of fluorescence intensities (Fig. 5, *A* and *B*). Images show nascent fibrils stained in green extending outward from cells visible by DAPI staining. At both 4 and 8 h time points, 25, 50, and 100 μg/ml heparin treatment increased nascent fibril assembly showing a concentration-dependent effect up to a 2.5- to 3-fold increase with 100 μg/ml heparin (Fig. 5*B*). Dextran sulfate or hyaluronic acid was not able to substitute for heparin in promoting nascent fibril formation (Fig. S3). Furthermore, nascent FN fibril assembly was significantly reduced in EXT1 knockdown fibroblasts (Fig. 5, *C* and *D*). However, this deficiency was completely rescued by treatment with 25 or 100 μg/ml heparin, showing equivalent levels of nascent fibril assembly between heparin-treated knockdown and mock cells. Therefore, HS is required for nascent FN fibril assembly and exogenous heparin promotes this process, even in cells that produce HS.

**Figure 6:**
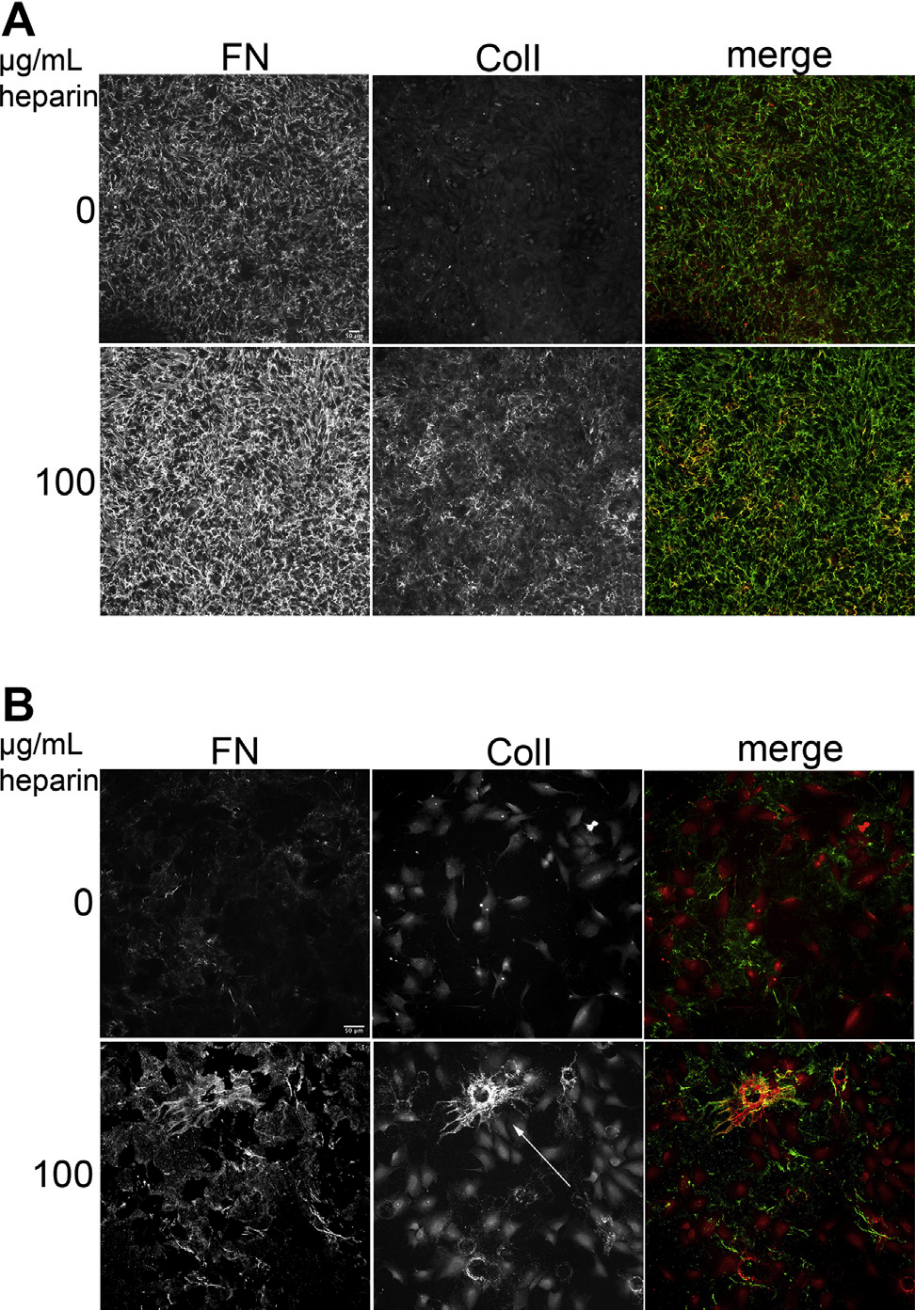
**Type I collagen fibrils increase with the addition of heparin.** Cells grown as in Figure 5 to confluence (24 h, *A*) or to subconfluence (12 h, *B*) along with 50 μg/ml ascorbic acid ±100 μg/ml heparin. Cells were fixed and stained with anti-human FN antibody (*green*) and anti-type I collagen antibody (Col1, *red*). Scale = 50 μm. *A* and *B*, representative images are shown for each antibody and for merged FN and type I collagen staining from three independent experiments.

Interestingly, along with FN, type I collagen staining was significantly increased in confluent NIH 3T3 cells treated with heparin (Fig. 6*A*, fold change = 1.61 ± 0.09, n = 3, *p* < 0.05). Furthermore, in subconfluent cell cultures, approximately 1.5% of cells grown in the presence of heparin assembled nascent collagen fibrils, which were not detected in the untreated condition (Fig. 6*B*). Collagen fibrils in both confluent and subconfluent cultures colocalized with FN fibrils. These findings demonstrate that heparin stimulation of FN fibril assembly also promotes type I collagen fibril formation.

**Figure 7:**
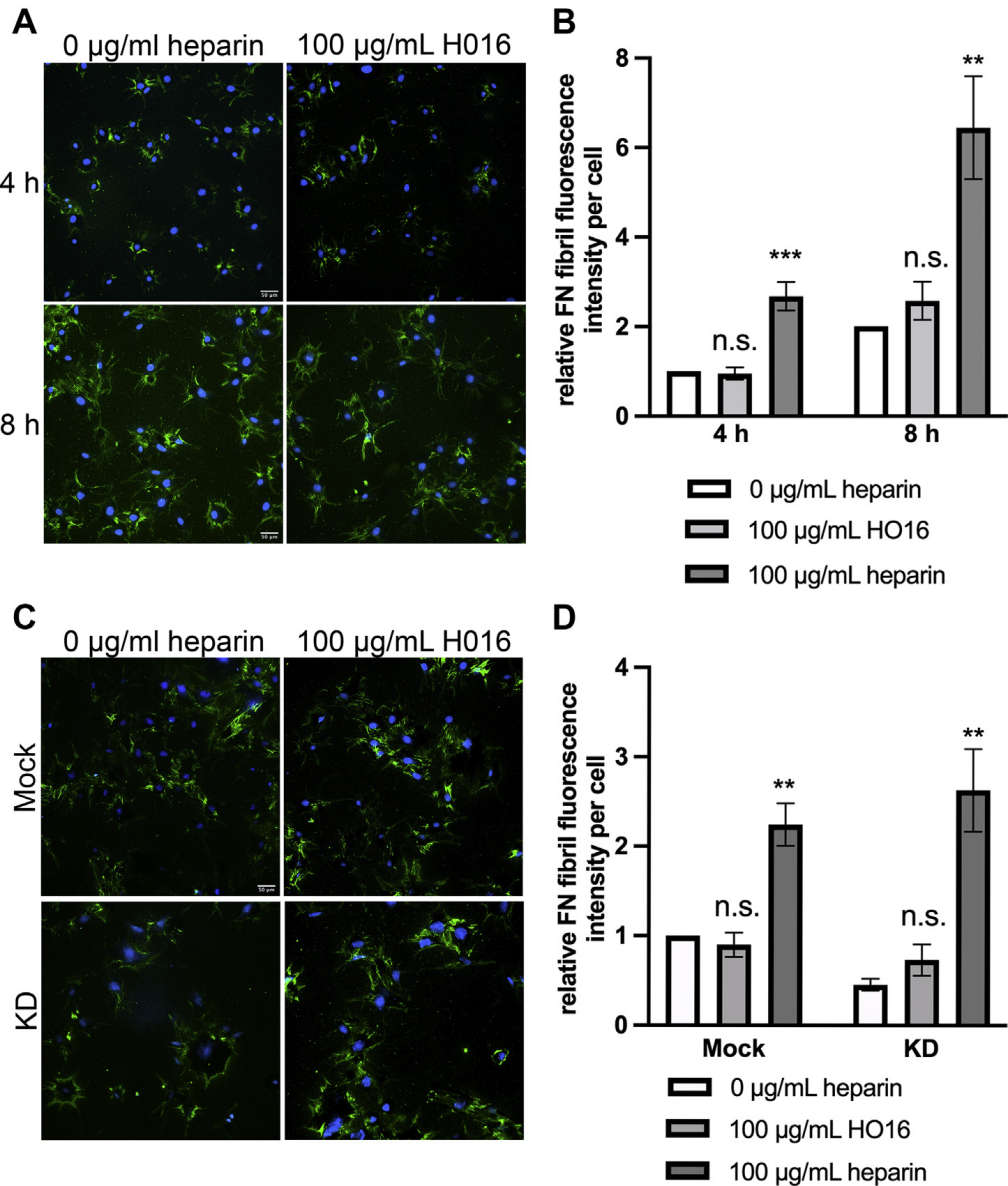
**Truncated heparin does not increase nascent fibril assembly.** NIH 3T3 (*A*) or mock and KD (*C*) cells were grown and analyzed as in Figure 5. 100 μg/ml heparin (60–70 saccharides) or HO16 (16 saccharides long) was added. The molarity of binding sites for FN is equivalent for the same mass of different-sized heparins. Representative images are shown for cells fixed and stained with anti-human FN antibody hFN7.1 (*green*) and DAPI (*blue*). Scale = 50 μm. FN fibril fluorescence intensity per cell ± HO16 or heparin was quantified for cells at 4 and 8 h as described in Figure 5 and expressed relative to 4 h–0 heparin treatment set to 1 (*B*). Mock and KD cells at 8 h were normalized to Mock cells–0 heparin treatment set to 1 (*D*). 100 μg/ml heparin bars for each condition in *B* and *D* are taken from Figure 5, which was part of the same experiments. Significance was determined for each treatment compared with 0 heparin in the same group. n.s. = not significant, ∗∗*p* < 0.01, ∗∗∗*p* < 0.001. Three (*A* and *B*) and four (*C* and *D*) independent experiments.

The heparin used to rescue the EXT1 deficiency in fibril formation is 60 to 70 saccharides long and, because it can bind ∼4 FN HepII domains simultaneously (11), could act to recruit and concentrate FN molecules. If this suggestion is correct, shorter heparin chains that bind fewer FNs would be deficient in promoting nascent fibril assembly. We used HO16, a 16-saccharide heparin chain, and HO30, with 30 saccharides, sufficient to bind one or two FN HepII domains (13, 25), respectively. No significant increase in nascent FN fibril staining was detected on cells treated with 100 μg/ml HO16 compared with untreated NIH 3T3 cells (Fig. 7, *A* and *B*). Likewise, HO16 treatment was not able to rescue nascent fibril formation by EXT1 knockdown fibroblasts (Fig. 7, *C* and *D*). No significant differences were detected between treatments with HO16 and HO30 in these experiments (data not shown). The truncated heparin results show that binding between heparin and one or two FN subunits is not sufficient to stimulate nascent fibril assembly. We conclude that more than two FN molecules must be localized by a single heparin chain to generate a detectable change in nascent fibrils.

## Discussion

The HepII domain of FN, containing the primary HS/heparin-binding site, has been implicated in matrix assembly, and our previous studies with CHO-677 cells that lack HS showed a role for this GAG in formation of DOC-insoluble fibrils, a late stage in the assembly process (13). CHO cells do not produce FN but can assemble a matrix if provided with exogenous FN (26). Here we show that NIH 3T3 fibroblasts that naturally assemble a dense FN matrix are similarly dependent on HS. The production of HS, the number of FN matrix fibrils, and the formation of DOC-insoluble matrix were all significantly decreased with knockdown of EXT1, one of the enzymes required to synthesize HS. Type I collagen fibrillogenesis was also severely reduced. Analysis of HS at the earliest stages in assembly identified a deficiency in formation of matrix assembly sites and nascent fibrils. Although matrix assembly sites were initiated, their growth was stunted in the absence of HS, indicating a role for HS in promoting FN binding at these sites. Subsequent formation of nascent FN fibrils was also decreased. That HS is the critical missing component was confirmed by rescue of nascent fibril assembly with exogenous heparin, which was only effective if it was sufficiently long to bind multiple FN molecules. From these results, we conclude that HS has an early and important role in matrix formation by gathering FN molecules near matrix assembly sites to promote nascent fibril assembly.

Soluble heparin was able to rescue nascent fibril formation by EXT1 knockdown cells indicating that HS does not have to be surface-bound in order to participate in these early steps of assembly. The rescue depended on heparin chain length since binding of truncated heparins to FN did not rescue FN fibrillogenesis. A longer heparin chain binding multiple FN molecules simultaneously could act as a bridge to aid in FN-FN interactions and facilitate fibrillogenesis (13). HS proteoglycans have been shown to exhibit such scaffold-like behavior by bringing growth factors into close proximity of their receptors (27), and several secreted proteins utilize HS as a bridge to oligomerize (28). HS also tethers proteins to localize and regulate the availability of morphogens and growth factors during development (28). A previous study has shown that the length and flexibility of the HS chain allow bound proteins to translocate within the pericellular space while continuing to stay localized near the cell surface, thereby influencing the distribution of signaling proteins such as fibroblast growth factor 2 (29). This scenario could also apply to nucleation of FN assembly by allowing HS-bound FN molecules to sample the cell surface until they encounter and are immobilized at integrin clusters characteristic of matrix assembly sites. The resulting increase in FN concentration at these sites would facilitate fibrillogenesis. The suggestion that HS/heparin is required for assembly site-specific localization at the cell surface is supported by our finding that heparin did not promote binding to cells in suspension. Furthermore, the ability of soluble heparin to rescue shows that this effect is independent of any protein-specific mechanism. HS/heparin can affect FN fibrillogenesis differently depending on cellular conditions. For example, exogenous heparin inhibited FN matrix assembly by HT1080 fibrosarcoma cells (5), which produce much less endogenous FN than NIH 3T3 fibroblasts. Apparently, the effects of heparin depend on the size of the pool of FN available for binding indicating that matrix assembly is affected by the level of synthesis of both HS and FN.

Loss-of-function mutations in EXT1 and EXT2, the enzymes responsible for HS synthesis, cause HME, an autosomal dominant disorder that is characterized by osteochondromas (30). Collagen I is necessary for proper bone development (17), and chondrocytes from HME patients show abnormal collagen production and deposition (31). Interestingly, type I collagen staining was significantly increased and colocalized with FN in heparin-treated cells and was greatly reduced in EXT1 knockdown cells, which is consistent with studies showing that FN provides a scaffold for type I collagen assembly and processing (21). It is therefore possible that defects in FN matrix caused by reduced or ablated HS production in HME play a pivotal role in the early stages of chondrogenesis leading to chondromas and other bone defects.

Both ECM-localized and transmembrane HSPGs are known to bind to FN and participate in many FN-dependent cell activities (1, 27). The effects of soluble heparin on matrix assembly do not implicate any particular HSPG in this process raising the possibility that several different HSPGs could contribute to assembly depending on the cells' microenvironment. For example, transmembrane syndecan-2 and -4 have demonstrated roles in cell binding to FN (32, 33). The extracellular HSPG perlecan also associates with FN (12) and is produced by NIH 3T3 fibroblasts (K. E. H., unpublished observations). HS chains on perlecan could facilitate FN association with newly formed matrix assembly sites. Interestingly, there is a decrease in perlecan in the matrix of EXT1-associated osteochondromas (34), supporting a connection between HSPGs, FN, and the etiology of HME. While specific HSPGs might be critical in assembly, our findings demonstrate a more general effect of HS/heparin on FN matrix assembly.

With these findings, we propose that HS/heparin increases the efficiency of matrix assembly initiation by localizing and retaining FN at nascent matrix assembly sites, thereby increasing its local concentration to drive the assembly process forward. We did not detect differences in fluorescence intensity of focal adhesion proteins paxillin or vinculin with or without heparin treatment (B. M. L., unpublished observations) indicating that heparin acts downstream of integrin clustering. By binding multiple FN molecules simultaneously, HS/heparin promotes FN-FN interactions. As a result of this early stimulation of matrix assembly site and nascent fibril formation, HS promotes the assembly of a robust FN matrix and enhances collagen fibrillogenesis. Finally, our model also suggests that HS’s effect on FN matrix assembly can be generalized beyond our EXT1 knockdown system. We predict that perturbations in the length or sulfation of HS chains will likely broadly affect ECM assembly by altering the efficiency of matrix assembly site growth, FN recruitment, and the subsequent deposition of ECM proteins. We find support for this prediction in previous work from our lab, which shows that reduced sulfation of proteoglycans with knockdown of diastrophic dysplasia sulfate transporter (DTDST) greatly decreases FN matrix assembly (5). Mutations in DTDST cause a variety of skeletal disorders (35), possibly due to loss of a foundational FN matrix. As both DTDST and EXT1’s effects can be viewed as perturbations of the HS chains, we believe our model highlights a new general mechanism for HS-dependent FN matrix assembly.

## Experimental procedures

### Cell culture, reagents, and antibodies

NIH 3T3 mouse fibroblasts (ATCC) were maintained in complete medium—Dulbecco’s Modified Eagle’s Medium (DMEM; Life Technologies) with 10% Bovine Calf Serum (BCS; Hyclone Laboratories). The cells were negative for *mycoplasma* contamination.

Fibronectin was purified from either frozen human plasma or frozen rat plasma *via* gelatin-Sepharose affinity chromatography as previously described (36). Recombinant 70 kD was purified from High 5 insect cells infected with a baculovirus expressing the rat N-terminal 70 kD region of FN (37) and subsequently biotinylated with EZ-Link Sulfo-NHS-Biotin (Thermo Fisher). Heparin sodium salt from porcine intestinal mucosa (Grade I-A, ≥180 USP U/mg) was obtained from Sigma. Shorter heparin H016 (MW = 4650) was from Iduron (Galen Laboratory Supplies).

Primary antibodies used in this study were rabbit anti-FN III_1–6_ antiserum (R184) (21), mouse anti-human FN monoclonal antibody (hFN7.1) (DSHB, U. Iowa), rabbit anticollagen I polyclonal antibody (PA2140-2, Boster Biological Technology), and mouse anti-heparan sulfate antibody (F58-10E4) from Amsbio. Antiglyceraldehyde 3-phosphate dehydrogenase (GAPDH) polyclonal antibody (Cell Signaling) was used to assess protein loading on immunoblots. Secondary antibodies were HRP-goat anti-rabbit IgG, HRP-goat anti-mouse IgG (Thermo Fisher Scientific), and Alexa Fluor 488-goat anti-mouse IgG and Alexa Fluor 568-goat anti-rabbit IgG (Invitrogen).

### EXT1 siRNA transfection of NIH 3T3 cells

NIH 3T3 cells were seeded at 150,000 cells per well in a 6-well dish in complete medium. After 24 h, cells were transfected with 200 nM EXT1 ONTarget Plus SmartPool siRNA reagent (Dharmacon) and 4 μl Lipofectamine 3000 (Invitrogen) in 1 ml Opti-MEM Reduced Serum Medium (Gibco). Mock transfected cells were treated with 4 μl lipofectamine 3000 in 1 ml Opti-MEM. After 4 h at 37 °C, 0.5 ml of complete medium was added and cells were incubated for 24 h at 37 °C. The transfection medium was removed and replaced with 2 ml complete medium. Cells were trypsinized and replated ∼18 h later for all assays.

### RNA extraction and qPCR

RNA from mock-treated and EXT1 knockdown cells was isolated 2 to 5 days posttransfection with TRIzol reagent (Invitrogen) followed by clean-up with a RNeasy column (Qiagen) (38). cDNA was prepared from 1 μg of total RNA using Protoscript II Reverse Transcriptase (NEB). SYBR Green PCR Master Mix (Applied Biosystems) was used with 400 nM of each primer. The qPCR reactions were run in duplicate in a Stratagene MX3000P thermocycler. Reaction conditions were as follows: 2 min at 50 °C and 10 min at 95 °C for one cycle, then 15 s at 95 °C, 30 s at 60 °C, and 30 s at 72 °C for 40 cycles. GAPDH was used for normalization. Data analysis was performed using MxPro qPCR software (Agilent Technologies). Primers were: forward EXT1 - 5′ GGATTGTTCGTCCTACCGCA 3′, reverse EXT1 - 5′ TTCAACACTGGCTGGGACTG 3′; forward GAPDH - 5′ AGCCTCGTCCCGTAGACAAA 3′, reverse GAPDH - 5′ GGCTTCCCGTTGATGACAAG 3′; forward FN - 5′ AAGGCTGGATGATGGTGGACTG 3′, reverse FN - 5′ TGAAGCAGGTTTCCTCGGTTG 3′.

### Cell lysis and immunoblotting

For analysis of matrix assembly, NIH 3T3 cells were seeded at 12 × 10^4^ cells per well in a 24-well dish and grown for 24 to 48 h. Samples for type I collagen analysis were supplemented with 50 μg/ml ascorbic acid in the media to enhance collagen processing and production. Cells were lysed in deoxycholate (DOC) buffer, and DOC-soluble and -insoluble fractions were isolated as previously described (39).

Proteins were separated on 5%, 6%, or 10% polyacrylamide–SDS gels along with Precision Plus Protein Standards (Bio-Rad) and transferred to nitrocellulose membranes. Incubations were performed in buffer A (25 mM Tris-HCl, pH 7.5, 150 mM NaCl, 0.1% Tween-20). Antibodies were used at the following dilutions: R184 (1:50,000), PA2140-2 anti-collagen I (0.15 μg/ml), and GAPDH (1:10,000). Secondary antibodies were diluted 1:10,000 in buffer A. Blots were developed with SuperSignal West Pico PLUS Chemiluminescent Substrate (Thermo Scientific). After development, densitometry was performed on scanned films with Adobe Photoshop Software. Signals in the linear range were normalized to GAPDH.

### Immunofluorescence microscopy

For matrix analyses, cells were grown in a 24-well plate on coverslips in complete medium with or without heparin as indicated. For collagen staining, cells were seeded in complete medium supplemented with 50 μg/ml ascorbic acid to induce collagen assembly and, in the indicated experiments, human FN ± 100 μg/ml heparin was also added. Cells either subconfluent or at confluence were washed, fixed, and stained for matrix fibrils as described (26). For HS analyses, subconfluent mock or knockdown NIH 3T3 cells, CHO-K1, and CHO-677 cells were fixed and stained. Primary antibodies were used at the following dilutions: R184 (1:100), hFN7.1 (1:100), F58-10E4 (1:100), and PA2140-2 anti-collagen I (1:500). Alexa Fluor 488 goat anti-mouse and Alexa Fluor 568 goat anti-rabbit were used at 1:500. DAPI (1:500) and Texas Red-X Phalloidin (1:100) were included where indicated. Coverslips were mounted in ProLong Gold anti-fade reagent. Images were captured using a Nikon Eclipse Ti microscope with a Hamamatsu C10600 ORCA-R2 digital camera and analyzed using Nikon Elements. Mean fluorescence intensities were measured in six random fields of view for each condition using ImageJ software. Images were adjusted equivalently for presentation using ImageJ software.

### Assays for FN matrix assembly sites and nascent fibril assembly

1 × 10^4^ cells were grown in a 24-well plate on glass coverslips precoated with a solution containing 10 μg/ml rat plasma FN in complete medium with the indicated amounts of heparin/HO16 added. For analysis of nascent FN fibrils, 25 μg/ml human plasma FN was added to the medium. At each time point, cells were fixed and stained with hFN7.1 antibody and Alexa Fluor 488 goat anti-mouse IgG. To quantify FN fibril fluorescence intensity, the mean background fluorescence intensity from the FN coat was measured in ImageJ and then subtracted from the mean fluorescence intensity of the entire image. The FN fibril fluorescence intensity was then divided by the number of nuclei within the image to normalize for variation in cell density. The average FN fibril fluorescence per cell of seven different fields of view was calculated for each condition, and the average of three separate experiments is reported. For analysis of matrix assembly sites, 1 h before cell fixation and staining, the culture medium was replaced with media containing 5 μg/ml biotinylated 70 kD, which was detected with Streptavidin Alexa Fluor 568 conjugate. Cells were also stained with anti-HS antibody and Alexa Fluor 488 goat anti-mouse IgG secondary antibody. Anti-HS and 70 kD fluorescence signals were measured in ImageJ. To quantify average HS and 70 kD fluorescence intensity per cell, a perimeter was drawn around individual cells using the freehand selections tool, and the total fluorescence intensity within that perimeter was measured. Then, the background fluorescence intensity within the perimeter was subtracted from the total fluorescence intensity. Average fluorescence intensity was calculated from at least 70 cells per experiment, and the average of three experiments was reported.

### Statistical analysis

Results are reported as the mean ± standard error for a minimum of three independent experiments. Statistical analyses were performed using an unpaired two-tailed Student’s *t* test, with *p* < 0.05 considered statistically significant.

## Data availability

All data are contained within the manuscript.

## Supporting information

This article contains supporting information.

## Conflict of interest

The authors declare that they have no conflicts of interest with the contents of this article.
