# Supplementary material for: Heparan sulfate is necessary for the early formation of nascent fibronectin and collagen I fibrils at matrix assembly sites
Source: J Biol Chem. 2021 Dec 7;298(1):101479. doi: 10.1016/j.jbc.2021.101479 (PMC8801470; doi:10.1016/j.jbc.2021.101479)
Supplement: Supplemental Figures S1–S3 and Tables S1, S2 [file mmc1.pdf]

Heparan sulfate is necessary for the early formation of nascent fibronectin and collagen I fibrils at matrix assembly sites

Katherine E. Hill, Benjamin M. Lovett, and Jean E. Schwarzbauer

List of material included:

Figure S1
Figure S2
Figure S3
Table S1
Table S2

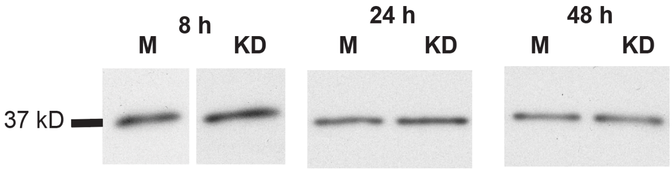

Figure S1: DOC-soluble cell lysates were isolated at the indicated times. Samples were separated on a 10% polyacrylamide-SDS gel, transferred and immunoblotted with an anti-GAPDH antiserum. These DOC-soluble cell lysates are from the same experimental replicate as the DOC-insoluble lysates shown in Figure 2.

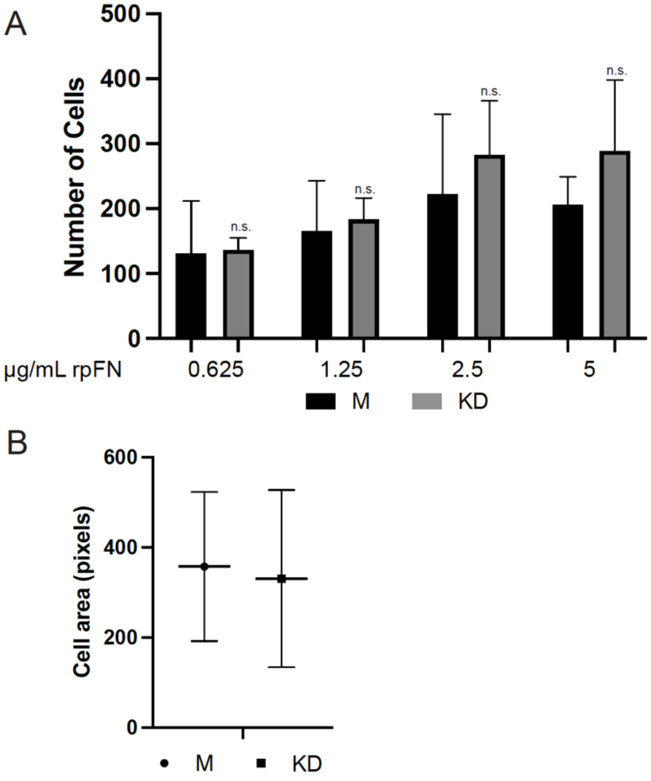

Figure S2: Decreased HS does not affect cell attachment to a FN substrate

(A) Coverslips were pre-coated with varying concentrations of rat plasma FN overnight at 4° C and then blocked in 1% BSA/PBS for 30 min at room temperature. 5 x 10^4 EXT1 knockdown and mock treated cells were plated on the coverslips with serum and allowed to attach for 2 h. Cells were then fixed, and actin was visualized with phalloidin for counting. Attached cells were counted in 12 fields of view for each condition. Error bars represent SEM. Values are a result of 2 independent experiments.

(B) EXT1 knockdown and mock treated cells were attached to coverslips coated with 2.5 μg/mL of rat plasma FN with serum for 2 h as described above. Cells were then fixed, and actin was visualized with phalloidin for cell shape. In Image J, a perimeter was drawn around individual cells using the freehand selections tool and then the cell area was measured. Cell area was measured for a minimum of 70 cells per condition. Error bars represent SEM. Values are a result of 2 independent experiments.

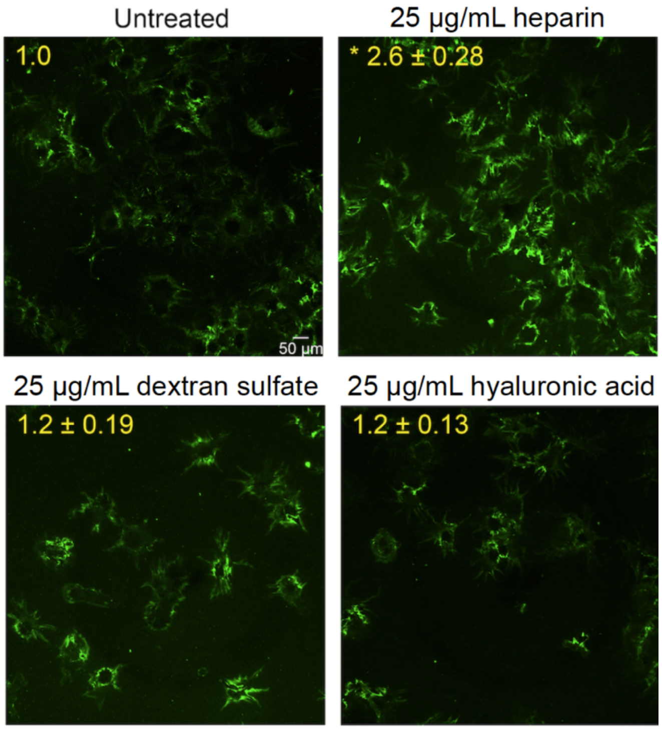

Figure S3: NIH 3T3 cells were grown on rat FN-coated coverslips in medium containing 25 µg/mL human FN and the indicated GAG treatment. Cells were fixed and stained with anti-human FN-specific antibody hFN7.1 (green). Scale = 50 µm. Representative images are shown for each condition. Indicated numbers are the average fold change in FN fibril fluorescence intensity per cell ± SEM relative to the untreated condition. FN fibril fluorescence was calculated as in Figure 5 and as described in the experimental procedures. Significance was determined for each treatment compared to untreated. *$p < 0.05$, n.s. = not significant.

Table S1. HS fluorescence intensity

2 h	Mock-treated			EXT-1 KD			
**Exp. #**	**Mean (A.U)**	**SD (A.U)**	**N**	**Low-HS Mean (A.U)**	**High-HS Mean (A.U**	**High: Low**	**N**
1	4.4	1.8	52	0.6	3.7	6.3	95
2	6.3	3.3	68	0.9	4.8	5.2	68
3	1.4	0.9	42	0.1	0.5	8.7	53
4	6.9	3.6	40	0.8	6.5	8.1	31
Avg.	4.8					7.1	
**4 h**	**Mock-treated**			**EXT-1 KD**			
**Exp. #**	**Mean (A.U)**	**SD (A.U)**	**N**	**Low-HS Mean (A.U)**	**High-HS Mean (A.U)**	**High: Low**	**N**
1	10.0	5.3	46	0.8	8.2	10.9	106
2	13.0	6.5	33	1.5	12.0	8.0	46
3	1.0	0.7	20	0.1	0.9	11.5	43
4	9.7	4.5	22	1.5	9.2	6.1	30
Avg.	8.4					9.1	

Table S1. HS fluorescence intensity statistics of mock-treated and EXT-1 knockdown cells at 2 and 4 h measured in arbitrary units (A.U) for 4 separate experiments.

Table S2. 70 kD fluorescence intensity

2 h	Mock-treated	EXT-1 KD		
Exp. #	Mean (A.U)	Low-HS mean (A.U)	High-HS mean (A.U)	High: Low
1	0.6	0.4	0.6	1.5
2	0.6	0.6	0.9	1.7
3	0.5	0.3	0.6	2.0
4	0.4	0.2	0.5	2.4
Avg.	0.5			1.9
**4 h**	**Mock-treated**	**EXT-1 KD**		
**Exp. #**	**Mean (A.U)**	**Low-HS mean (A.U)**	**High-HS mean (A.U)**	**High: Low**
1	0.9	0.6	1.0	1.8
2	0.8	0.7	1.3	1.8
3	0.7	0.5	0.9	2.0
4	0.9	0.4	0.8	2.0
Avg.	0.8			1.9

Table S2. 70 kD fluorescence intensity statistics of mock-treated and EXT-1 knockdown cells at 2 and 4 h measured in arbitrary units (A.U) for 4 separate experiments.
